# 1,2-Bis[(2,2′:6′,2′′-terpyridin-4′-yl)­oxy]ethane

**DOI:** 10.1107/S1600536812030292

**Published:** 2012-07-07

**Authors:** Varvara I. Nikolayenko, Matthew P. Akerman, Craig D. Grimmer, Desigan Reddy

**Affiliations:** a School of Chemistry and Physics, University of KwaZulu-Natal, Private Bag X01, Scottsville 3209, Pietermaritzburg, South Africa

## Abstract

The title compound, C_32_H_24_N_6_O_2_, has an inversion centre located at the mid-point of the central C—C bond of the diether bridging unit. The terminal pyridine rings are canted relative to the central pyridine ring, with dihedral angles of 12.98 (6) and 26.80 (6)°. The maximum deviation from the eight-atom mean plane, defined by the two bridging O and C atoms and the central pyridine ring, is 0.0383 (10)° for the N atom.

## Related literature

For the structure of the un-substituted 2,2′:6′,2′′-terpyridine compound, see: Bessel *et al.* (1992 ▶). For the structure of the precursor to the title compound, 4′-chloro-2,2′:6′,2′′-ter­pyridine, see: Beves *et al.* (2006 ▶). For the structure of 1,4-bis­[(2,2′:6′,2′′-terpyridin-4′-yl)­oxy]butane, see: Akerman *et al.* (2011 ▶). For the structure of 1,6-bis­[(2,2′:6′,2′′-terpyridin-4′-yl)­­oxy]hexane, see: Nikolayenko *et al.* (2012 ▶). For a full review of functionalized 2,2′:6′,2′′-terpyridine complexes, see: Fallahpour (2003 ▶); Heller & Schubert (2003 ▶). For a comprehensive summary of platinum(II) terpyridines, see: Newkome *et al.* (2008 ▶). For the structure of bis­(2,2′:6′,2′′-terpyrid­yl) ether, see: Constable *et al.* (1995 ▶). For the syntheses and structures of related bis­(terpyridine) structures linked by an alk­oxy spacer, see: Constable *et al.* (2006 ▶). For the syntheses of diol-bridged terpyridines, see: Constable *et al.* (2005 ▶); Van der Schilden (2006 ▶).
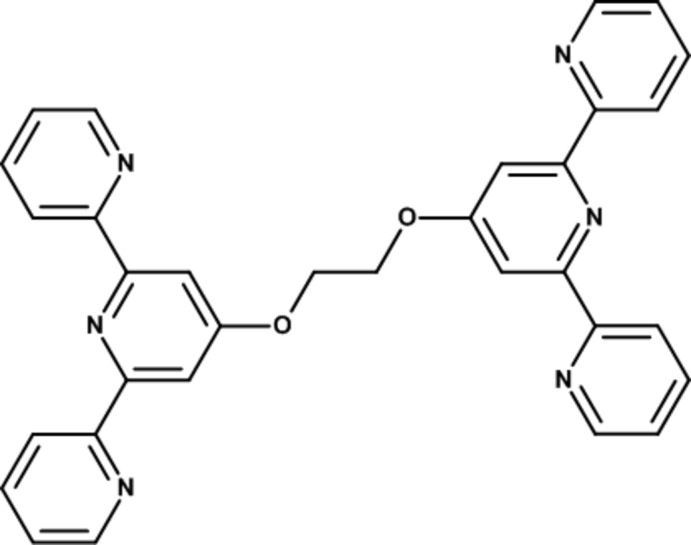


## Experimental

### 

#### Crystal data

C_32_H_24_N_6_O_2_
*M* *_r_* = 524.58
Triclinic, 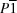

*a* = 6.2576 (6) Å
*b* = 10.0851 (9) Å
*c* = 10.2388 (9) Å
α = 93.850 (6)°
β = 98.760 (6)°
γ = 102.468 (5)°
*V* = 620.20 (10) Å^3^
*Z* = 1
Mo *K*α radiation
μ = 0.09 mm^−1^
*T* = 100 K
0.20 × 0.10 × 0.05 mm

#### Data collection

Bruker APEXII CCD diffractometer
Absorption correction: multi-scan (*SADABS*; Bruker, 2010 ▶) *T*_min_ = 0.982, *T*_max_ = 0.996
8371 measured reflections
4459 independent reflections
3364 reflections with *I* > 2σ(*I*)
*R*_int_ = 0.041

#### Refinement

*R*[*F* ^2^ > 2σ(*F* ^2^)] = 0.062
*wR*(*F* ^2^) = 0.182
*S* = 1.04
4459 reflections
181 parameters
H-atom parameters constrained
Δρ_max_ = 0.77 e Å^−3^
Δρ_min_ = −0.27 e Å^−3^

### 

Data collection: *APEX2* (Bruker, 2010 ▶); cell refinement: *SAINT-Plus* (Bruker, 2010 ▶); data reduction: *SAINT-Plus*; program(s) used to solve structure: *SHELXL97* (Sheldrick, 2008 ▶); program(s) used to refine structure: *SHELXL97* (Sheldrick, 2008 ▶); molecular graphics: *WinGX* (Farrugia, 1999 ▶); software used to prepare material for publication: *publCIF* (Westrip, 2010 ▶).

## Supplementary Material

Crystal structure: contains datablock(s) I, global. DOI: 10.1107/S1600536812030292/fj2578sup1.cif

Structure factors: contains datablock(s) I. DOI: 10.1107/S1600536812030292/fj2578Isup2.hkl

Supplementary material file. DOI: 10.1107/S1600536812030292/fj2578Isup3.cml

Additional supplementary materials:  crystallographic information; 3D view; checkCIF report

## Figures and Tables

**Fig. 1.**
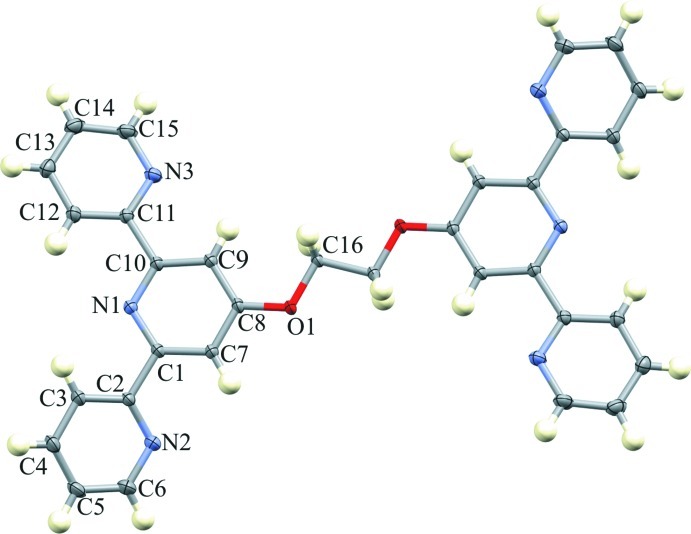
The molecular structure of the title compound. Displacement ellipsoids are drawn at 50% probability level.

**Fig. 2.**
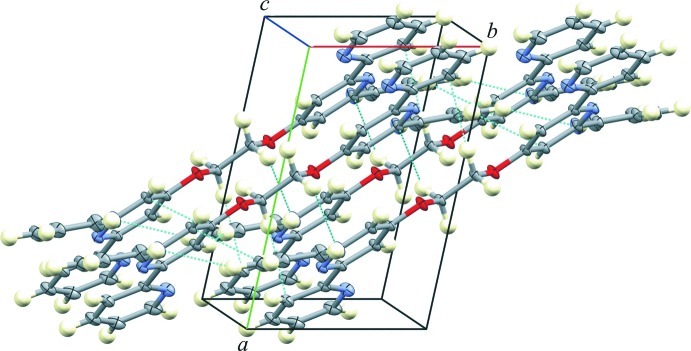
A view of packing of the title compound.

## supplementary crystallographic information

### Comment

The title compound is the last in a series of ligands developed in an effort to boost multifunctional activity. Coordination of these ligands to platinum(II) should enable covalent binding of DNA through both metal centres, thus increasing the number of adducts formed. Furthermore the presence of the flexible diol derived linkage will provide the complex with the potential to engage in long range interactions with DNA.

The ligand crystallized in the triclinic space group P1, with a half molecule in the asymmetric unit and *Z* = 1. Crystallographically imposed inversion symmetry relates the two halves of the ligand to one another. The inversion centre is located at the mid-point of the diol linkage unit. The three pyridine rings adopt a *trans, trans* conformation. The same configuration is observed in the other ligands of this series with butyl and hexyl diol linkages (Akerman *et al.*, 2011; Nikolayenko *et al.*, 2012). The parent compound 4'-chloro-2,2':6',2''-terpyridine (Beves *et al.* 2006), and uncoordinated terpyridine ligands in general show the same configuration.

The central pyridine ring of the terpyridine moeity is in the same plane as the bridging diol chain. The terminal pyridine rings of the terpyridine ligand are, however, canted relative to the central pyridine ring. The C7—C6—C5—N1 torsion angle is 25.9 (2)° while the C9—C10—C11—N3 torsion angle is 11.9 (2)° (refer to Fig. 1 for the atom numbering scheme). The large torsion angle of the pyridine ring containing N1 is seemingly to allow for hydrogen bonding between the pyridine nitrogen atom N1 and the pyridine hydrogen atom H3 of an adjacent molecule. This hydrogen bond links the molecules into an infinite, one-dimensional chain (Fig. 2). The hydrogen bonded chain is co-linear with the *a*-axis. The hydrogen bond lengths and bond angles are summarized in Table 1. Although the hydrogen bond length does not necessarily correlate linearly with bond strength, due to packing constraints, the interaction is relatively long and it is therefore likely to be a weak interaction. There is no indication of meaningful π–π or C—H···π interactions in the lattice, which are often observed in terpyridine structures (Beves *et al.* 2006).

### Experimental

The title compound was prepared by an adaptation of a previously described method (Van der Schilden, 2006; Constable *et al.*, 2005). Ethanediol (1.13 mmol) was added to a suspension of ground potassium hydroxide (6.69 mmol) in DMSO (30 ml). The solution was heated to reflux for 1 h after which 4'-chloro-2,2':6'2''-terpyridine (2.23 mmol) was added. The mixture was again brought to reflux for an additional 24 h. After cooling to room temperature, the brown mixture was added to cold water (100 ml). The resulting off-white precipitate was collected, rinsed with cold ethanol and air dried. Single crystals were grown by slow liquid diffusion of n-hexane into a chloroform solution of the compound.

### Refinement

All non-hydrogen atoms were located in the difference Fourier map and refined anistropically. The positions of all hydrogen atoms were calculated using the standard riding model of *SHELX97*. with C—H(aromatic) and C—H(methylene) distances of 0.93 Å and *U*_iso_ = 1.2*U*_eq_.

### Crystal data

**Table:**

C_32_H_24_N_6_O_2_	*Z* = 1
*M**_r_* = 524.58	*F*(000) = 274
Triclinic, *P*1	*D*_x_ = 1.405 Mg m^−^^3^
Hall symbol: -P 1	Mo *K*α radiation, λ = 0.71073 Å
*a* = 6.2576 (6) Å	Cell parameters from 3364 reflections
*b* = 10.0851 (9) Å	θ = 2.0–32.8°
*c* = 10.2388 (9) Å	µ = 0.09 mm^−^^1^
α = 93.850 (6)°	*T* = 100 K
β = 98.760 (6)°	Needle, colourless
γ = 102.468 (5)°	0.20 × 0.10 × 0.05 mm
*V* = 620.20 (10) Å^3^	

### Data collection

**Table:**

Bruker APEXII CCD diffractometer	4459 independent reflections
Radiation source: fine-focus sealed tube	3364 reflections with *I* > 2σ(*I*)
Graphite monochromator	*R*_int_ = 0.041
ω and φ scans	θ_max_ = 32.8°, θ_min_ = 2.0°
Absorption correction: multi-scan (*SADABS*; Bruker, 2010)	*h* = −9→8
*T*_min_ = 0.982, *T*_max_ = 0.996	*k* = −15→15
8371 measured reflections	*l* = −15→15

### Refinement

**Table:**

Refinement on *F*^2^	Primary atom site location: structure-invariant direct methods
Least-squares matrix: full	Secondary atom site location: difference Fourier map
*R*[*F*^2^ > 2σ(*F*^2^)] = 0.062	Hydrogen site location: inferred from neighbouring sites
*wR*(*F*^2^) = 0.182	H-atom parameters constrained
*S* = 1.04	*w* = 1/[σ^2^(*F*_o_^2^) + (0.1018*P*)^2^ + 0.0609*P*] where *P* = (*F*_o_^2^ + 2*F*_c_^2^)/3
4459 reflections	(Δ/σ)_max_ = 0.001
181 parameters	Δρ_max_ = 0.77 e Å^−^^3^
0 restraints	Δρ_min_ = −0.27 e Å^−^^3^

### Special details

**Table:**

Geometry. All s.u.'s (except the s.u. in the dihedral angle between two l.s. planes) are estimated using the full covariance matrix. The cell s.u.'s are taken into account individually in the estimation of s.u.'s in distances, angles and torsion angles; correlations between s.u.'s in cell parameters are only used when they are defined by crystal symmetry. An approximate (isotropic) treatment of cell s.u.'s is used for estimating s.u.'s involving l.s. planes.
Refinement. Refinement of *F*^2^ against ALL reflections. The weighted *R*-factor *wR* and goodness of fit *S* are based on *F*^2^, conventional *R*-factors *R* are based on *F*, with *F* set to zero for negative *F*^2^. The threshold expression of *F*^2^ > 2σ(*F*^2^) is used only for calculating *R*-factors(gt) *etc*. and is not relevant to the choice of reflections for refinement. *R*-factors based on *F*^2^ are statistically about twice as large as those based on *F*, and *R*-factors based on ALL data will be even larger.

### Fractional atomic coordinates and isotropic or equivalent isotropic displacement parameters (Å^2^)

**Table:**

	*x*	*y*	*z*	*U*_iso_*/*U*_eq_	
C1	0.8540 (2)	0.33313 (15)	0.60520 (14)	0.0283 (3)	
H1	0.7963	0.3499	0.6837	0.034*	
C2	1.0660 (2)	0.30976 (14)	0.61813 (14)	0.0269 (3)	
H2	1.1508	0.3100	0.7034	0.032*	
C3	1.1514 (2)	0.28615 (14)	0.50440 (14)	0.0250 (3)	
H3	1.2977	0.2721	0.5101	0.030*	
C4	1.0195 (2)	0.28323 (13)	0.38123 (13)	0.0208 (3)	
H4	1.0724	0.2648	0.3013	0.025*	
C5	0.80907 (19)	0.30792 (12)	0.37783 (12)	0.0166 (2)	
C6	0.66344 (19)	0.30803 (12)	0.24834 (11)	0.0162 (2)	
C7	0.5019 (2)	0.38379 (12)	0.24126 (12)	0.0174 (2)	
H7	0.4845	0.4371	0.3173	0.021*	
C8	0.36599 (19)	0.37913 (11)	0.11862 (12)	0.0164 (2)	
C9	0.3930 (2)	0.29829 (12)	0.01032 (12)	0.0175 (2)	
H9	0.2993	0.2913	−0.0733	0.021*	
C10	0.56189 (19)	0.22744 (11)	0.02768 (12)	0.0163 (2)	
C11	0.5964 (2)	0.14184 (12)	−0.08808 (12)	0.0176 (2)	
C12	0.7865 (2)	0.09098 (13)	−0.08388 (13)	0.0216 (3)	
H12	0.8959	0.1077	−0.0057	0.026*	
C13	0.8136 (2)	0.01537 (14)	−0.19586 (14)	0.0263 (3)	
H13	0.9440	−0.0183	−0.1966	0.032*	
C14	0.6474 (3)	−0.01030 (14)	−0.30669 (14)	0.0286 (3)	
H14	0.6605	−0.0627	−0.3845	0.034*	
C15	0.4620 (3)	0.04248 (14)	−0.30107 (14)	0.0277 (3)	
H15	0.3471	0.0234	−0.3765	0.033*	
C16	0.07750 (19)	0.45488 (12)	−0.01226 (11)	0.0167 (2)	
H16A	−0.0069	0.3610	−0.0461	0.020*	
H16B	0.1703	0.4909	−0.0778	0.020*	
N1	0.72577 (19)	0.33330 (12)	0.48743 (11)	0.0235 (2)	
N2	0.69872 (17)	0.23327 (10)	0.14323 (10)	0.0170 (2)	
N3	0.4356 (2)	0.11876 (11)	−0.19559 (11)	0.0231 (2)	
O1	0.21257 (15)	0.45611 (9)	0.11416 (8)	0.0196 (2)	

### Atomic displacement parameters (Å^2^)

**Table:**

	*U*^11^	*U*^22^	*U*^33^	*U*^12^	*U*^13^	*U*^23^
C1	0.0317 (7)	0.0380 (7)	0.0155 (6)	0.0149 (6)	−0.0030 (5)	−0.0009 (5)
C2	0.0312 (7)	0.0294 (6)	0.0178 (6)	0.0113 (5)	−0.0085 (5)	0.0004 (5)
C3	0.0227 (6)	0.0284 (6)	0.0231 (6)	0.0116 (5)	−0.0060 (5)	0.0004 (5)
C4	0.0200 (5)	0.0256 (6)	0.0177 (6)	0.0111 (4)	−0.0015 (4)	0.0004 (4)
C5	0.0185 (5)	0.0175 (5)	0.0138 (5)	0.0074 (4)	−0.0016 (4)	0.0011 (4)
C6	0.0174 (5)	0.0184 (5)	0.0135 (5)	0.0077 (4)	−0.0004 (4)	0.0020 (4)
C7	0.0195 (5)	0.0191 (5)	0.0145 (5)	0.0095 (4)	−0.0006 (4)	0.0007 (4)
C8	0.0170 (5)	0.0170 (5)	0.0164 (5)	0.0085 (4)	−0.0004 (4)	0.0025 (4)
C9	0.0199 (5)	0.0192 (5)	0.0141 (5)	0.0092 (4)	−0.0015 (4)	0.0012 (4)
C10	0.0181 (5)	0.0166 (5)	0.0148 (5)	0.0075 (4)	−0.0001 (4)	0.0007 (4)
C11	0.0218 (5)	0.0166 (5)	0.0150 (5)	0.0080 (4)	0.0003 (4)	0.0005 (4)
C12	0.0251 (6)	0.0205 (5)	0.0201 (6)	0.0110 (4)	−0.0005 (4)	−0.0003 (4)
C13	0.0348 (7)	0.0236 (6)	0.0247 (7)	0.0154 (5)	0.0060 (5)	0.0007 (5)
C14	0.0449 (8)	0.0234 (6)	0.0188 (6)	0.0127 (6)	0.0046 (5)	−0.0028 (5)
C15	0.0399 (8)	0.0247 (6)	0.0162 (6)	0.0103 (5)	−0.0044 (5)	−0.0037 (5)
C16	0.0175 (5)	0.0184 (5)	0.0148 (5)	0.0089 (4)	−0.0022 (4)	0.0015 (4)
N1	0.0245 (5)	0.0319 (6)	0.0155 (5)	0.0141 (4)	−0.0016 (4)	−0.0004 (4)
N2	0.0190 (5)	0.0179 (4)	0.0146 (5)	0.0081 (4)	−0.0007 (3)	0.0007 (3)
N3	0.0292 (6)	0.0233 (5)	0.0158 (5)	0.0103 (4)	−0.0034 (4)	−0.0019 (4)
O1	0.0219 (4)	0.0248 (4)	0.0145 (4)	0.0155 (3)	−0.0033 (3)	−0.0008 (3)

### Geometric parameters (Å, º)

**Table:**

C1—N1	1.3431 (16)	C9—H9	0.9500
C1—C2	1.385 (2)	C10—N2	1.3404 (14)
C1—H1	0.9500	C10—C11	1.4891 (16)
C2—C3	1.381 (2)	C11—N3	1.3437 (14)
C2—H2	0.9500	C11—C12	1.3895 (17)
C3—C4	1.3928 (16)	C12—C13	1.3865 (18)
C3—H3	0.9500	C12—H12	0.9500
C4—C5	1.3879 (16)	C13—C14	1.3859 (19)
C4—H4	0.9500	C13—H13	0.9500
C5—N1	1.3393 (17)	C14—C15	1.384 (2)
C5—C6	1.4902 (15)	C14—H14	0.9500
C6—N2	1.3457 (15)	C15—N3	1.3354 (17)
C6—C7	1.3889 (15)	C15—H15	0.9500
C7—C8	1.3972 (15)	C16—O1	1.4318 (13)
C7—H7	0.9500	C16—C16^i^	1.502 (2)
C8—O1	1.3564 (13)	C16—H16A	0.9900
C8—C9	1.3849 (16)	C16—H16B	0.9900
C9—C10	1.3945 (15)		
			
N1—C1—C2	123.44 (13)	N2—C10—C11	117.52 (10)
N1—C1—H1	118.3	C9—C10—C11	118.88 (10)
C2—C1—H1	118.3	N3—C11—C12	122.67 (11)
C3—C2—C1	118.57 (11)	N3—C11—C10	116.21 (10)
C3—C2—H2	120.7	C12—C11—C10	121.12 (10)
C1—C2—H2	120.7	C13—C12—C11	118.78 (11)
C2—C3—C4	118.90 (12)	C13—C12—H12	120.6
C2—C3—H3	120.6	C11—C12—H12	120.6
C4—C3—H3	120.6	C14—C13—C12	118.95 (12)
C5—C4—C3	118.52 (12)	C14—C13—H13	120.5
C5—C4—H4	120.7	C12—C13—H13	120.5
C3—C4—H4	120.7	C15—C14—C13	118.25 (12)
N1—C5—C4	123.13 (10)	C15—C14—H14	120.9
N1—C5—C6	116.44 (10)	C13—C14—H14	120.9
C4—C5—C6	120.42 (11)	N3—C15—C14	123.72 (12)
N2—C6—C7	123.58 (10)	N3—C15—H15	118.1
N2—C6—C5	116.82 (9)	C14—C15—H15	118.1
C7—C6—C5	119.60 (10)	O1—C16—C16^i^	105.29 (11)
C6—C7—C8	117.85 (10)	O1—C16—H16A	110.7
C6—C7—H7	121.1	C16^i^—C16—H16A	110.7
C8—C7—H7	121.1	O1—C16—H16B	110.7
O1—C8—C9	123.90 (10)	C16^i^—C16—H16B	110.7
O1—C8—C7	116.52 (10)	H16A—C16—H16B	108.8
C9—C8—C7	119.58 (10)	C5—N1—C1	117.40 (11)
C8—C9—C10	118.00 (10)	C10—N2—C6	117.30 (9)
C8—C9—H9	121.0	C15—N3—C11	117.59 (11)
C10—C9—H9	121.0	C8—O1—C16	116.69 (9)
N2—C10—C9	123.59 (10)		
			
N1—C1—C2—C3	0.4 (2)	C9—C10—C11—C12	167.31 (12)
C1—C2—C3—C4	−1.6 (2)	N3—C11—C12—C13	1.2 (2)
C2—C3—C4—C5	1.72 (19)	C10—C11—C12—C13	−178.02 (12)
C3—C4—C5—N1	−0.60 (19)	C11—C12—C13—C14	−1.9 (2)
C3—C4—C5—C6	179.03 (11)	C12—C13—C14—C15	0.8 (2)
N1—C5—C6—N2	−154.39 (12)	C13—C14—C15—N3	1.2 (2)
C4—C5—C6—N2	25.96 (17)	C4—C5—N1—C1	−0.62 (19)
N1—C5—C6—C7	25.91 (17)	C6—C5—N1—C1	179.74 (12)
C4—C5—C6—C7	−153.74 (12)	C2—C1—N1—C5	0.7 (2)
N2—C6—C7—C8	1.52 (19)	C9—C10—N2—C6	2.55 (18)
C5—C6—C7—C8	−178.80 (11)	C11—C10—N2—C6	−178.40 (11)
C6—C7—C8—O1	−178.82 (11)	C7—C6—N2—C10	−3.38 (18)
C6—C7—C8—C9	1.27 (18)	C5—C6—N2—C10	176.93 (10)
O1—C8—C9—C10	178.08 (11)	C14—C15—N3—C11	−1.9 (2)
C7—C8—C9—C10	−2.02 (18)	C12—C11—N3—C15	0.7 (2)
C8—C9—C10—N2	0.09 (19)	C10—C11—N3—C15	179.90 (13)
C8—C9—C10—C11	−178.94 (11)	C9—C8—O1—C16	−1.95 (18)
N2—C10—C11—N3	168.96 (11)	C7—C8—O1—C16	178.14 (10)
C9—C10—C11—N3	−11.94 (18)	C16^i^—C16—O1—C8	178.97 (12)
N2—C10—C11—C12	−11.79 (18)		

Symmetry code: (i) −*x*, −*y*+1, −*z*.
